# A Superhydrophobic, Antibacterial, and Durable Surface of Poplar Wood

**DOI:** 10.3390/nano11081885

**Published:** 2021-07-23

**Authors:** Xinyu Wu, Feng Yang, Jian Gan, Zhangqian Kong, Yan Wu

**Affiliations:** 1 Department of Wood Products Engineering, College of Furnishings and Industrial Design, Nanjing Forestry University, Nanjing 210037, China; wuxinyu@njfu.edu.cn (X.W.); agumpJ@njfu.edu.cn (J.G.); kongzhangqian@njfu.edu.cn (Z.K.); 2 Co-Innovation Center of Efficient Processing and Utilization of Forest Resources, Nanjing Forestry University, Nanjing 210037, China; 3 Beijing Institute of Fashion Technology, Fashion Accessory Art and Engineering College, Beijing 100029, China; yangfeng@bift.edu.cn

**Keywords:** superhydrophobicity, wood modification, antimicrobial properties, durability

## Abstract

The silver particles were grown in situ on the surface of wood by the silver mirror method and modified with stearic acid to acquire a surface with superhydrophobic and antibacterial properties. Fourier transform infrared spectroscopy (FTIR), X-ray diffraction (XRD), and X-ray energy spectroscopy (XPS) were used to analyze the reaction mechanism of the modification process. Scanning electron microscopy (SEM) and contact angle tests were used to characterize the wettability and surface morphology. A coating with a micro rough structure was successfully constructed by the modification of stearic acid, which imparted superhydrophobicity and antibacterial activity to poplar wood. The stability tests were performed to discuss the stability of its hydrophobic performance. The results showed that it has good mechanical properties, acid and alkali resistance, and UV stability. The durability tests demonstrated that the coating has the function of water resistance and fouling resistance and can maintain the stability of its hydrophobic properties under different temperatures of heat treatment.

## 1. Introduction

As a natural material, wood is one of the most important materials for structural construction, furniture, energy, and aerospace industries [1]. However, the negative characteristics of wood, such as water absorption, UV degradation, and poor resistance to microbial action, have limited the application of wood [2,3]. Therefore, special treatment of the wood surface to obtain hydrophobicity is necessary to prolong the service life of wood and give greater application to the value of wood [4,5,6,7,8,9].

Because superhydrophobic materials have special wettability [10], it is widely used in the fields of self-cleaning [11,12], antifouling [13], antisepsis [14,15], and oil–water separation [16,17]. The methods of preparing superhydrophobic wood include the etching method [18], the vapor deposition method [19], and the layer-by-layer self-assembly method [20]. When water droplets contact the surface, an air pad will be formed in the groove of the micro-nano rough structure on the superhydrophobic surface. Thus, a solid–liquid-gas three-phase interface is formed, and the contact surface between water droplets and the surface is increased to prevent water droplets from infiltrating the surface [21,22,23]. Superhydrophobic surfaces are usually obtained in two steps: the first is to use nanoparticles such as ZnO [24], SiO_2_ [19], TiO_2_ [25], and CaCO_3_ to generate rough structures on the surface of the medium [26,27]; the second is to modify a rough surface with a low-surface energy substance [28,29]. At present, it is more common to attach nanoparticles to the wood surface by polymer and treat them with the substrate of low surface energy; however, due to the low adhesion between nanoparticles and wood, superhydrophobic wood often has poor mechanical properties. To improve the bonding force between interfaces and enhance the mechanical wear resistance of the wood surface, nanoparticles can be generated in situ on the wood surface through chemical reactions [13,30]. At the same time, considering that wood contains cellulose, hemicellulose, lignin, starch, sugar, and other components, it is a hotbed for microbial growth [31,32]. When the temperature and humidity and other environmental conditions are appropriate, these microorganisms will attach to the wood for a large number of growth and reproduction, resulting in wood decay, discoloration, and moldy, which influence the strength, toughness, and permeability of wood [6,33,34,35]. It also poses a threat to the life and health of the users [36,37,38]. Therefore, antibacterial treatment of wood is needed to maintain the properties of wood and prevent the spread of disease [39].

At present, the preparation of antimicrobial wood methods includes siloxane antifouling coating, copolymer, polymer, and other methods to inhibit the growth of bacteria; however, these polymer-based materials are often easily removed from the wood, showing poor mechanical properties. Therefore, it is necessary to develop a wood coating with high mechanical durability and antimicrobial properties [40]. The organic–inorganic mixed coating prepared by the sol-gel method can substitute for polymer-based antimicrobial coating, which is usually prepared from metallic alkanes or functional alkanes as precursors. It has environmental benefits and ensures strong mechanical properties and antifouling performance [41,42]. Silver nanoparticles have a strong antibacterial effect, which is since silver ions can inhibit the synthesis of enzymes on the extra-mercaptan groups of proteins on the microbial membrane, and eventually lead to the death of cells [43]. At the same time, silver nanoparticles have a smaller particle size and larger specific surface area, so they can dissolve in solution and release silver ions, thus achieving stronger antibacterial properties [44]. In general, the physical and chemical properties of silver nanoparticles, such as size, shape, or surface characteristics, can be controlled by chemical substances such as polymer, metal–ion interaction, or various reducing agents to construct functional surfaces [45]. Duan [46] prepared the wood with superhydrophobic antibacterial properties by self-polymerization of dopamine and hydrophobic modification of copper nanoparticles on the surface of the wood with fluoro silane. The coating showed good resistance to acid or alkali corrosion and good mechanical properties. Gao [47] treated wood with sodium hydroxide and silver nitrate, then reduced nano-silver ions on the wood surface with glucose and modified with fluoro silane to prepare a kind of superhydrophobic and oil-phobic wood with electrical conductivity. These methods of preparation of superhydrophobic wood used the chemical reaction directly on the wood surface to grow nanoparticles, guarantee the superhydrophobic coating and the cohesive strength between wood, thus showed excellent mechanical properties. This method will be beneficial to expand the application forms of superhydrophobic wood, and has a high application value in the fields of self-cleaning, biomedicine, and electronic information.

Here, we successfully deposited silver particles on the wood surface through the reaction of silver ammonia solution and sodium hydroxide with a simple silver mirror reaction [48]. First, the poplar wood was pretreated with NaOH solution to make the surface of it negatively charged. It absorbed Ag(NH_3_)_2_^+^ after being impregnated with a silver ammonia complex solution. Ag(NH_3_)_2_^+^ was reduced to Ag particles in situ on the wood surface by formaldehyde reduction. After that, the treated wood was modified by stearic acid with a long-chain alkyl group [49] to obtain a superhydrophobic surface with a contact angle of 158.7°. The modified surface has waterproof, fouling, and antibacterial properties, and stable superhydrophobic properties under mechanical wear cycle, acid and alkali solution, ultraviolet light, and different temperatures of heat treatment.

## 2. Materials and Methods

### 2.1. Materials

The poplar wood (*Populus tomentosa*) with 20 mm in length and width, 2 mm in thickness, was provided by Yixuan Material Co. Ltd., Dongguan, China. Its moisture content was 9.5% and the absolute dry density was 284.5 kg/m^3^. Sodium hydroxide (NaOH, 96%), ammonia (NH_3_·H_2_O, 28%), acetic acid (CH_3_COOH, 99.5%) were purchased from Nanjing Chemical Reagent Co., Ltd., Nanjing, China. The silver nitrate solution (AgNO_3_) was from Guangzhou Kyle Chemical Co., Ltd., Guangzhou, China. Formaldehyde solution (CH_2_O) was supplied by Shanghai Jiuyi Chemical Reagent Co., Ltd., Shanghai, China. The anhydrous ethanol (99.7%) was from Sinopharm Chemical Reagent Co., Ltd., Shanghai, China, and the stearic acid (C_18_H_36_O_2_) was from Tianjin Chemical Technology Co., Ltd., Tianjin, China.

### 2.2. Methods

The poplar wood used in this experiment was ultrasonically cleaned (Misonix, Inc., New York, NY, USA) in anhydrous ethanol for 30 min and distilled water for 30 min and dried at 60 °C in a constant temperature blast drying oven (Shangchen Instrument Co., Ltd., Shaoxin, China). Then the dried wood was impregnated in sodium hydroxide solution with a concentration of 0.1 M for 12 h. After taking it out, the poplar was cleaned by ultrasound with distilled water for 15 min and dried at room temperature. Then 0.1 M silver nitrate and sodium hydroxide solution was arranged in a three-mouth flask. After mixing, ammonia water was added drop by drop, and it was constantly stirred until the solution becomes clear and transparent. The wood was impregnated in the solution of silver ammonia for one hour and then a certain amount of formaldehyde solution was added and stirred in the DF-101Z magnetic stirrer (Lichen Scientific Instrument Factory, Shanghai, China) with a constant temperature at 6 °C for 15 min to prepare the wood coated with silver (wood@Ag). After extraction, it was dried at 120 °C for 2 h, then was immersed in the ethanol mixture of 30 mL stearic acid (4 wt%) and acetic acid (0.08 M). After 4 min, it was taken out and dried at room temperature to obtain the poplar wood with the silver grafted with stearic acid (wood@Ag@SA).

### 2.3. Analysis of Reaction Mechanism

Vertex 80 V Fourier Transform Infrared Spectrometer (Brock Spectral Instruments GmbH, Karlsruhe, Germany) was used to analyze the chemical composition on the surface of the wood sample loaded with Ag (wood@Ag) and after modification of the stearic acid (wood@Ag@SA), with the wavenumber of 500–4000 cm^−1^. The surface chemical composition of wood@Ag and wood@Ag@SA, over the range of 30°−80°, was characterized by Axis Ultradld XRD (Rigaku Corporation, Osaka, Japan). The element composition and content of wood@Ag and wood@Ag@SA were characterized by Axis Ultradld XPS (Nippon Koji Co. Ltd., Tokyo, Japan). The scanning mode was CAE, and the spectrum was calibrated with the reference voltage of C 1s = 284.6 eV.

### 2.4. Surface Morphology and Wettability Analysis

A small piece of wood@Ag and wood@Ag@SA were pasted on the observation platform through the conductive adhesive and sprayed with gold. The surface morphology was observed under vacuum by Quanta 200 environmental scanning electron microscopy (FEI Corporation, New York, NY, USA) (Hong, Sunghwan). The water contact angles (WCAs) of wood@Ag@SA were measured by the Theta T200 optical contact Angle analyzer (Biolin Technologies GmbH, Gothenburg, Sweden). The droplet volume was 6 μL. The test was repeated three times on the surface of each sample to achieve the average value. Then, the water bouncing test was conducted and recorded, which is the process having droplets of water fall onto the contact surface until it left the surface.

### 2.5. Antimicrobial Test

The antibacterial activity of wood@Ag@SA was qualitatively evaluated by Staphylococcus aureus (ATCC 2592, Gram-positive) [50]. First, the nutrient agar was dissolved together with the nutrient broth in deionized water with a pH of 7. The mixture was then autoclaved with an empty petri dish at 121 °C for 30 min. The obtained agar medium was separated and cooled in laminar flow. Then 3,300,000 CFU/mL of bacteria were covered on the Petri dishes. Finally, the wood samples were placed in Petri dishes and cultured at 37 °C for 24 h. The bacterial colony count method was used to calculate according to Formula (1)

(1) $$R=\frac{\left(T_{0}-T_{1}\right)}{T_{0}}\times 100\%$$

where *T*_0_ is the number of bacteria in the blank sample; *T*_1_ is the number of bacteria in the plate of the tested sample. After 3 counts, the average value of the antibacterial rate *R* was obtained.

### 2.6. Stability Test

In the mechanical wear resistance test, with the force of 150 N, the surface of Ag@SA@wood was pressed by fingers, and then it experienced 50 times friction cycles at 9.8 KPa pressure, using 2000 mesh sandpaper on the surface of 100 cm reciprocating friction. It was continued to be scratched with a knife on the surface to form grid marks, with the distance between two adjacent marks around 2 mm [51]. Finally, the WCAs of wood@Ag@SA after the mechanical wear resistance test were recorded. It was impregnated in solutions after immersion in HCl solution (pH = 1) and NaOH solution (pH = 12) for 24 h, then it was washed with distilled water and dried at 85 °C to measure the WCAs of the surface. To test the UV stability of wood@Ag@SA, it was exposed to a 365 nm UV lamp for 6 h, and the wettability of its surface was tested.

### 2.7. Contaminant Resistance Test and Durability Testing

Common daily liquids such as tea, milk, coffee, and orange juice were dropped on the surface of wood@Ag@SA with a syringe to test its performance of antifouling [52]. To determine the water resistance of wood@Ag@SA, the original poplar wood and wood @Ag@SA were impregnated in deionized water and placed at room temperature for a while. The samples were taken out and dried at 85 °C for 1 h to record their weight every 5 days. The water absorption of the samples before and after modification was calculated by the formula (2)

(2) $$\mathrm{WA}\left(\%\right)=\left(m_{ai}-m_{bi}\right)/m_{bi}\times 100\%$$

where *m_ai_* is the weight after water absorption and *m_bi_* is the weight of dry wood. To discuss the heat resistance of wood@Ag@SA, it was kept in a constant temperature blast drying oven at the temperature of 20 °C, 40 °C, 60 °C, 80 °C, 80 °C, and 120 °C, for six hours, respectively, and the WCAs of it were tested.

## 3. Results

### 3.1. Analysis of Reaction Mechanism

#### 3.1.1. FTIR and XRD

**Figure 1:**
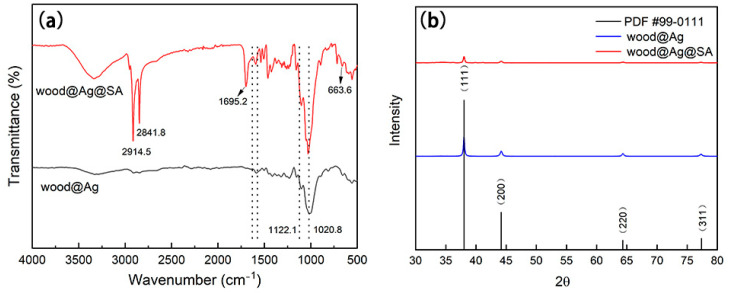
The infrared spectra (**a**) and X-ray diffraction spectra (**b**) of wood@Ag and wood@Ag@SA.

Figure 1a shows the infrared spectra of wood@Ag and wood@Ag@SA, in which the bending vibration of –CH_3_(–CH_2_–) was at 1456.6 cm^1,^ and the peak at 2841.8 cm^−1^ and 2914.5 cm^1^ was the stretching vibration of –CH_3_(–CH_2_–). It showed that the stearic acid had successfully modified wood@Ag. The vibration absorption peak of Ag-O appeared at 663.6 cm^−1^. The peak from 1020.8 cm^−1^ to 1120.1 cm^−1^ corresponded to the asymmetric stretching vibration peak of C–O in the CH_3_ (CH_2_)_16_COO– group. The absorption peak attributed to –COO– appeared at 1580–1620 cm^−1^, indicating that –COOH of stearic acid reacted chemically with Ag^+^ of wood@Ag to generate the –COOAg group. To effectively understand the chemical composition of wood@Ag and wood@Ag@SA, the crystal composition of the coating surface was tested by XRD. The results are shown in Figure 1b. The main peaks were 38.1°, 44.2°, 64.4°, and 77.4°, respectively. Therefore, it can be concluded that the main composition of the coating surface of wood@Ag and wood@Ag@SA was Ag. The results reflected that the purity of Ag on the surface of wood@Ag@SA was low and the crystallinity was poor.

#### 3.1.2. XPS

**Figure 2:**
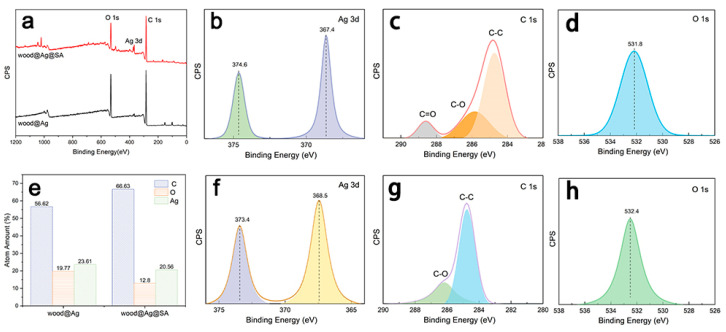
(**a**) The XPS survey of wood@Ag and wood@Ag@SA; (**b**) Ag 3d XPS spectra of wood@Ag; (**c**) C 1s XPS spectra of wood @Ag; (**d**) O 1s spectra of wood@Ag; (**e**) the atom amount distribution of wood@Ag and wood@Ag@SA; (**f**) Ag 3d XPS spectra of wood@Ag@SA; (**g**) C 1s XPS spectra of wood @Ag@SA; (**h**) O 1s spectra of wood@Ag@SA.

Figure 2 shows the XPS spectra of wood@Ag and wood@Ag@SA. The main elements were carbon, oxygen, and silver. As shown in Figure 2c, the peak at 284.7 eV belonged to C–C in stearic acid molecules, the peak at 285.8 eV was C–O, and the peak at 288.5 eV corresponded to C=O [53]. In Figure 2g, the peak at 286.1 eV belonged to C–O and the peak at 284.7 eV was distributed as C-C. After the modification of stearic acid, the surface of wood@Ag@SA had higher carbon content, lower oxygen content, and lower silver content. The carbon content of it increased from 56.62% to 66.63%, whereas the oxygen content decreased from 19.77% to 12.8%.

#### 3.1.3. Analysis of Modification Mechanism

**Figure 3:**
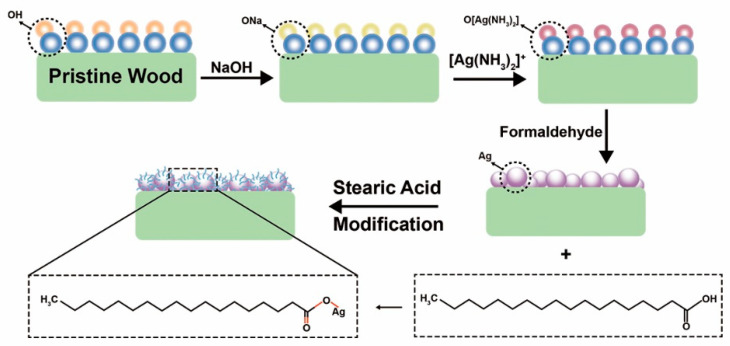
The modification mechanism of wood@Ag@SA.

Figure 3 reveals the modification mechanism of wood@Ag@SA. The pretreatment of sodium hydroxide formed OH^−^ on the wood surface. A mixture of sodium hydroxide and silver nitrate reacted with ammonia to form a solution of silver ammonia. Then, the wood absorbed Ag(NH_3_)_2_^+^ after being impregnated with the silver ammonia complex solution. Through the reduction of formaldehyde, Ag(NH_3_)_2_^+^ was reduced to Ag particles on the wood surface. Stearic acid acts as an amphiphilic ligand in the synthesis of Ag@SA because it has a carboxylic acid head group bound to the surface of Ag^+^ and a hydrophobic alkyl chain tail facing the external medium. Under the catalysis of acetic acid, the carboxylic acid of stearic acid reacted with silver ions, the H^+^ of –COOH was replaced by silver ions, and the hydrophobic group –CH_3_ with low surface energy was introduced on the surface of silver particles.

### 3.2. Surface Morphology and Wettability Analysis

**Figure 4:**
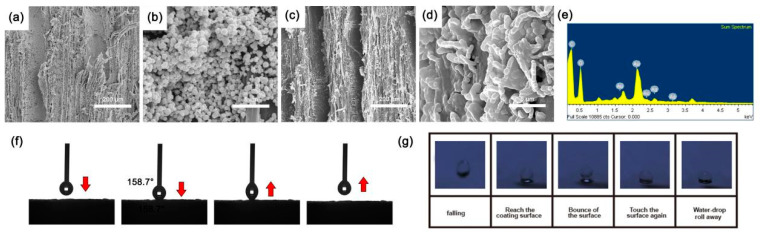
(**a**) SEM images of wood@Ag at 200 magnification; (**b**) SEM images of wood@Ag at 8000 magnification; (**c**) SEM images of wood@Ag@SA at 200 magnification; (**d**) SEM images of wood@Ag@SA at 8000 magnification and wood@Ag@SA; (**e**) EDS images of wood@Ag@SA; (**f**) WCA test of wood@Ag@SA; (**g**) water bouncing test of wood@Ag@SA.

Figure 4 reveals the surface morphology and wettability of wood@Ag@SA. As shown in Figure 4a, the fibers on the surface of poplar wood were uniformly loaded with a layer of Ag particles. As can be seen in Figure 4b, the Ag particles were spherical on the surface of the fibers of poplar wood, forming a rough structure. It can be observed in Figure 4c that after modification, the long-chain hydrophobic alkyl in the stearic acid molecule was successfully grafted to the wood surface. It can be observed from Figure 4d that the surface of the coating was distributed with coral-like papillae with a slender root connecting the dense particles to the matrix. This grooved structure avoided the agglomeration phenomenon between particles. Meanwhile, the introduction of long hydrophobic alkyl chains reduced the surface energy of the coating [54]. It can be observed in Figure 4e that the main elements on the surface of wood@Ag@SA were C, O, and Ag. Figure 4f is the test diagram of the contact angle between the surface and water wood@Ag@SA. The contact angle of water on the surface was 158.7°. The water droplets completely leave the surface without wetting, indicating that the surfaces were superhydrophobic. This was because the methyl-grafted silver particles form an air cushion between the coating and the water droplets, thus decreasing the contact area between the coating and the water drop, and achieving the superhydrophobic performance of wood@Ag@SA. The result of the water bouncing test was shown in Figure 4g. The process of droplets dropping from the beginning to the contact surface until they leave the surface shows the low viscoelasticity of water droplets and the surface [55].

### 3.3. Antimicrobial Test

**Figure 5:**
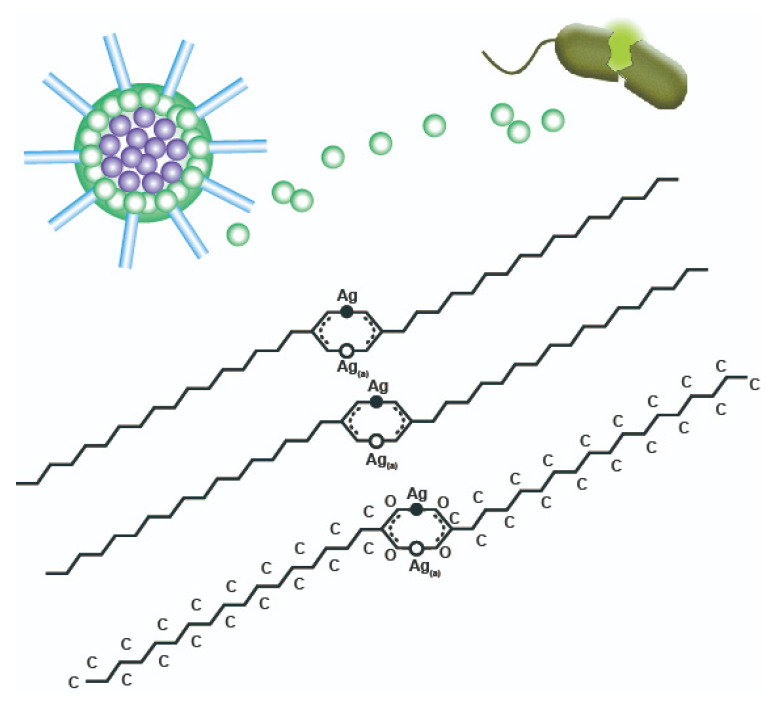
The antimicrobial mechanism of wood@Ag@SA.

As shown in Table 1, colony analysis was carried out on wood and liquefied wood to calculate the colony formation unit (CFU) [56]. It showed that the antimicrobial rate of wood@Ag@SA to Staphylococcus aureus could reach 99.9%, indicating that wood@Ag@SA has a good antibacterial effect. The antibacterial mechanism of the coating is introduced in Figure 5, showing the atomic arrangement and particle structure on the surface of wood@Ag@SA. The core of the particles was a silver atom, and the outer was a silver ion. Some of Ag^+^ grafted with hydrophobic alkyl, and the other part was free. The antibacterial properties of wood@Ag@SA were realized through its superhydrophobic properties and the antibacterial properties of Ag^+^. On the one hand, the superhydrophobicity of wood@Ag@SA can reduce the contact area between the coating and the bacterial solution, thus realizing the bacteriostatic effect. On the other hand, the Ag^+^ on the surface of wood@Ag@SA had a strong oxidation capacity, which could destroy the protein, lipid, and DNA of the cell through the catalytic effect of the cell enzyme, so that the cell produced metabolic disorders, thus losing activity. The silver ions on the surface of wood@Ag@SA could also bind with enzymes in donor sulfhydryl groups to release other ions in the cell, thus making it difficult for cellular enzymes to play their roles and causing bacterial inactivation [57].

### 3.4. Stability Test

**Figure 6:**
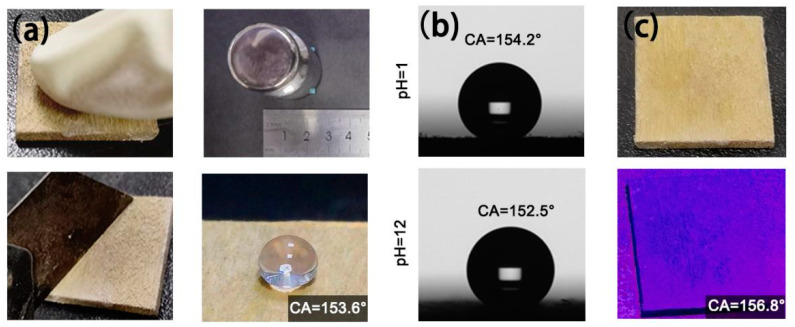
The WCAs of wood@Ag@SA after the mechanical wear resistance test (**a**) and after immersion in HCl solution (pH = 1) and NaOH solution (pH = 12) (**b**), and after exposure to a 365 nm UV lamp for 6 h (**c**).

As shown in Figure 6a, the contact angle of wood@Ag@SA was 153.6° after the mechanical wear resistance test. This was because the pretreatment of sodium hydroxide made the surface of wood be negatively charged and firmly combined with the Ag particles generated by the reaction of the silver mirror. It made the rough structures and low-surface substances on the surface of the wood remain stable after repeated friction or cutting, giving the coating strong mechanical stability [58]. Figure 6b shows the WCAs of wood@Ag@SA during the acid and alkali resistance test. After 500 h of impregnation in acidic solution, the WCA of it was 154.2°, and after 500 h of impregnation in alkaline solution, the WCAs was 152.5°, indicating that wood@Ag@SA has the certain corrosion resistance of acid and alkali solution. The prepared wood@Ag@SA also had a certain ability to resist ultraviolet radiation. In Figure 6c, after the coating was irradiated under the UV lamp for 72 h, the water droplets remain spherical on the surface of the sample. The test showed that the WCA of it is 156.8°, which proved that wood@Ag@SA has certain UV stability [59]. This was because the organic–inorganic hybrid process has generated –CH_3_ on the surface of wood@Ag@SA, which has certain stability and was difficult to degrade under ultraviolet radiation, so the low surface energy of the surface can be maintained.

### 3.5. Contaminant Resistance Test and Durability Test

**Figure 7:**
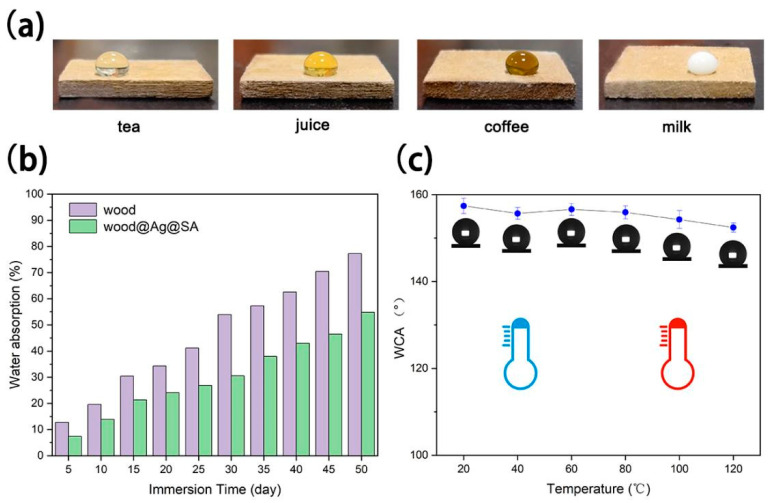
The images of different contaminants on the wood@Ag@SA (**a**) Changes of water adsorption for wood@Ag@SA as a function of time after immersion in water; (**b**) changes of WCA for wood@Ag@SA as a function of time after being treated in a constant temperature blast drying oven at the temperature of 20 °C, 40 °C, 60 °C, 80 °C, 80 °C, and 120 °C for six hours (**c**).

Figure 7a shows the contaminant resistance test of wood@Ag@SA, which shows that common domestic contaminants did not leave traces on the surface due to a certain water repellency of the surface. Figure 7b shows that with the increase of immersion days, the water absorption of wood and wood@Ag@SA shows an upward trend, while the water absorption of wood@Ag@SA was always lower than that of wood. When the immersion days were less than 30 days, the water absorption rate of wood@Ag@SA was almost constant. When the immersion days increased to 50 days, the water absorption of wood reached 77.2%, while wood@Ag@SA was 54.8%, which still maintained a low water absorption level. This indicated that the water repellant of wood@Ag@SA inhibited its absorption of water, thus reducing the possibility of shrinkage deformation and mildew in the moisture environment. It can be seen from Figure 7c that wood@Ag@SA can still maintain superhydrophobicity after treatment at various temperatures. After treatment at 120 °C for 6 h, the WCA on the surface of wood@Ag@SA was 152.4°, which proved that it has good heat resistance [25,60,61,62].

## 4. Conclusions

In this experiment, a convenient and effective method for preparing superhydrophobic and antimicrobial wood was proposed by the organic–inorganic mixing method. The Ag particles were prepared on a wood surface with the disposal of a silver ammonia complex solution and then reduced with formaldehyde. The Ag particles were coated with low surface energy stearic acid and further modified to obtain a superhydrophobic surface with a WCA of 158.7°. The formation mechanism and antimicrobial mechanism of the rough structure were analyzed. The surface morphology was characterized, and the stability test was carried out. The mechanical wear resistance, UV resistance, and acid and alkali resistance of wood@Ag@SA were tested. The results showed that it could maintain the superhydrophobic property in a harsh environment. The antifouling and water absorption tests showed that the modified wood was waterproof and fouling resistant. The poplar wood coating showed excellent properties in hydrophobicity, antimicrobial property, and durability, making it suitable for self-cleaning and biomedical applications.

## Figures and Tables

**Table 1:** The antimicrobial test of wood@Ag@SA.

Name of the Sample	Colony Forming Unit (CFU)	Antimicrobial Rate of Staphylococcus Aureus (%)
wood@Ag@SA	500	99.9

## Data Availability

Not applicable.
